# Leveraging Geometry to Enable High-Strength Continuum Robots

**DOI:** 10.3389/frobt.2021.629871

**Published:** 2021-02-18

**Authors:** Jake A. Childs, Caleb Rucker

**Affiliations:** Department Mechanical, Aerospace, and Biomedical Engineering, University of Tennessee - Knoxville, Knoxville, TN, United States

**Keywords:** soft robot, continuum robot, soft manipulation, soft robot actuation, soft robot analysis

## Abstract

Developing high-strength continuum robots can be challenging without compromising on the overall size of the robot, the complexity of design and the range of motion. In this work, we explore how the load capacity of continuum robots can drastically be improved through a combination of backbone design and convergent actuation path routing. We propose a rhombus-patterned backbone structure composed of thin walled-plates that can be easily fabricated via 3D printing and exhibits high shear and torsional stiffness while allowing bending. We then explore the effect of combined parallel and converging actuation path routing and its influence on continuum robot strength. Experimentally determined compliance matrices are generated for straight, translation and bending configurations for analysis and discussion. A robotic actuation platform is constructed to demonstrate the applicability of these design choices.

## 1 Introduction

**FIGURE 1:**
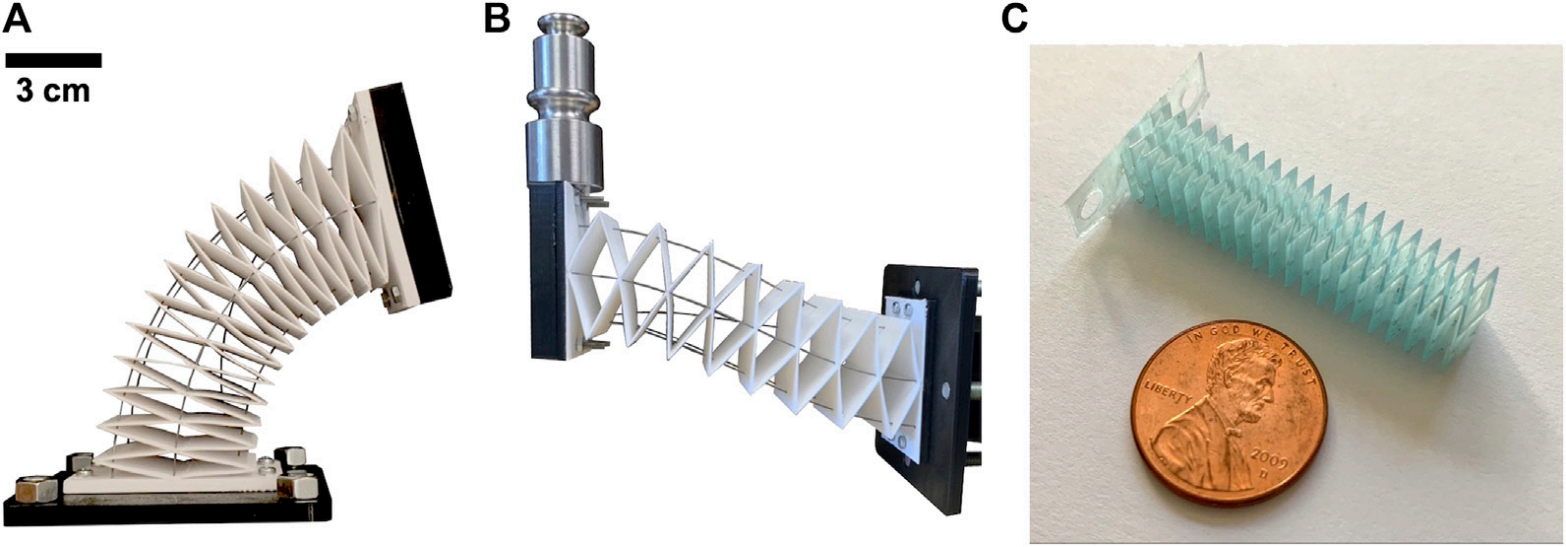
Our novel continuum robot utilizes a torsionally strong backbone and converging actuation routing **(A)**. This robot design has a high payload capacity **(B)** and can be fabricated at both large and small scales **(C)**.

Soft and continuum robots have great potential in a variety of applications, from small-scale surgical manipulators that can maneuver through confined pathways within the human body (Burgner-Kahrs et al., 2015) to large-scale arms that can interact with people and manipulate objects (Fathi et al., 2019; Nguyen et al., 2019). One of the current challenges in the design of soft and continuum robot is developing manipulators that have larger payload capacity. For slender continuum robots, there are at least two passive modes of deformation that limit robot strength in the presence of external loads. First, in near-straight configurations, an s-shape deformation mode typically occurs when a transverse load is applied [see Kim et al. (2014)]. Second, in curved configurations, out-of plane transverse loads cause significant deflection due to torsional deformation at the base and along the length, in addition to bending deflection. These deformations occur in part due to the under-constrained nature of typical continuum robot actuation, and in part due to insufficient torsional stiffness. In this paper, we propose to address both of these weaknesses through a combination of a novel flexible backbone design, and novel actuation routing paths along the backbone. The specific geometry of both these design choices helps to increase the load bearing capacity at the end effector without sacrificing actuatable range of motion. In particular, we use an extruded thin-plate rhombus pattern for the backbone that is lightweight, torsionally stiff, and simple to manufacture. Additionally we route the actuation rods in both parallel and converging paths to eliminate the main underactuated bending modes. This creates a 5-DOF robot, as shown in Figures 1 A,B, capable of both bending and translation motions with high output stiffness throughout its workspace. This robot concept can be fabricated at both large scales with conventional fused filament fabrication and at small scales with stereolithography as shown in Figure 1C.

The robotics research community has been investigating novel robot designs and advanced fabrication techniques to improve the payload capacity and performance of continuum robots. One way to improve strength is to design the backbone structures to have desired mechanical properties such as having a high torsional rigidity (corresponding to *GJ* for a beam, the product of shear modulus *G* and torsion constant *J*). Utilizing endo- and exo-skeleton designs has been shown to prevent buckling and improve overall strength (Fishman et al., 2017; Castledine et al., 2019). Bellows, which have high torsional rigidity in comparison with its flexural rigidity, have also been used in pneumatic (Drotman et al., 2017; Dämmer et al., 2019) and concentric-tube robots (Childs and Rucker, 2020) which can drastically improve the torsional strength of the overall robot. Origami-inspired structures show great promise as many of these designs can be tuned from geometric parameters and can be made on a wide range of scales (Liu et al., 2018; Rus and Sung, 2018; Santoso and Onal, 2020). Active elements such as granular jamming can be added to manipulators to actively control the stiffness of the robot (Jiang et al., 2012; Jiang et al., 2019). With these design strategies, improving the strength performance of continuum robots can be challenging without introducing adverse effects which can include an increase in the overall weight of the robot or complexity in fabrication and assembly.

In addition to backbone design, the actuation path routing can also be utilized to improve the strength of a continuum robot and/or increase its range of motion (Kim et al., 2017; Starke et al., 2017; Case et al., 2018) The majority of tendon- and rod-driven continuum robots utilize parallel actuation path routing in which the tendons or rods are parallel to the centerline of the backbone. It has been demonstrated that utilizing straight actuation paths that converge to each other or to the backbone center can significantly reduce translational compliance at the end-effector of the robot (Oliver-Butler et al., 2019), as well as altering the kinematics. Similar to the origami-inspired work cited above, our proposed backbone design also takes inspiration from origami, but it is simpler to fabricate since its geometry is based on simple linear extrusion of a planar pattern, making it amenable to 3D printing and other manufacturing methods. Also, our backbone’s degrees of freedom arise primarily due to plate bending and warping instead of only the conventional hinge degree of freedom of classical origami. Additionally we aim to show that a combination of parallel and convergent actuator routing paths increases payload capacity by eliminating passive degrees of freedom, while also enabling a 5-DOF workspace that includes purely translational motions.

We have structured our paper into two sections. In ‘section 2,’ we detail our methods for backbone design and determination of the mechanical properties of a prototype design from finite element analysis (FEA). We also detail the parallel and converging actuation routing selection and how it mitigates tip deflection from end-effector loads. We also discuss the robotic prototype used to validate our proposed high-strength design, and the setup used to experimentally determine the compliance matrix. In ‘section 3,’ we show our results for the mechanical properties of the backbone, the experimental compliance matrices, and payload demonstration.

## 2 Materials and Methods

### 2.1 Backbone Design With High Torsional Rigidity

**FIGURE 2:**
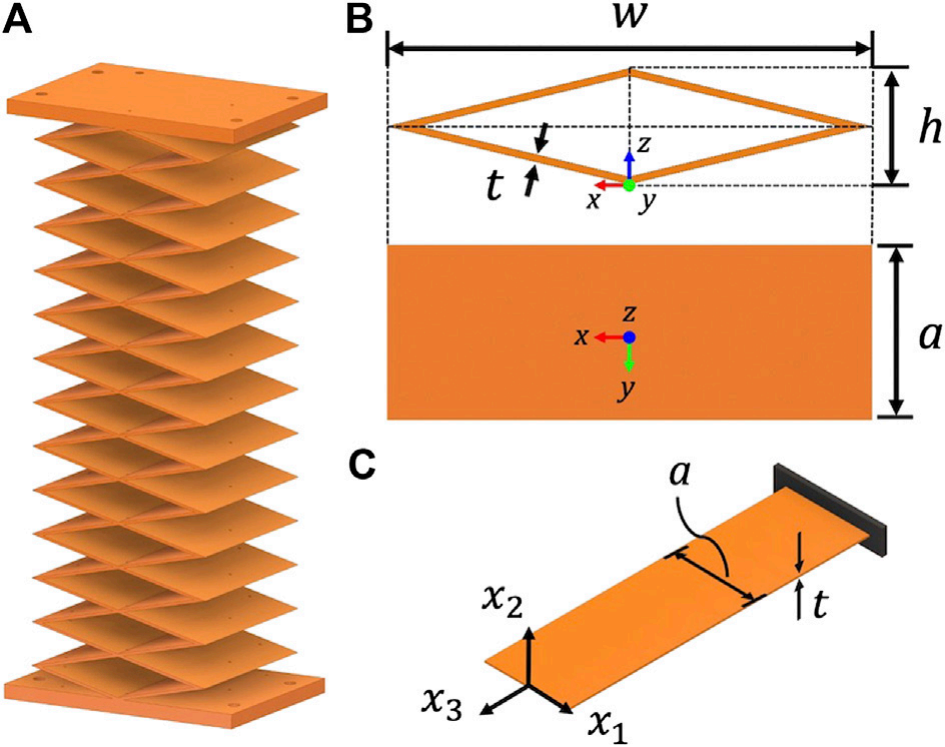
Backbone design with repeated rhombus sections **(A)**, a unit cell rhombus which is dictated by its width *w*, height *h*, wall thickness *t* and extruded depth *a*, **(B)** and a single plate model from the unit cell **(C)**.

As previously discussed, the backbone structure of continuum robots plays a key role in their bulk properties and performance. We have developed a simple backbone design, shown in Figure 2A, that uses a patterned unit cell. This unit cell can be geometrically described as a rhombus with an overall width *w*, height *h*, wall thickness *t* and extruded depth *a* as shown in Figure 2B. The modes of deformation that occur within this unit cell arise due to bending and warping from the individual plates that connect the structure into a rhombus. If we investigate the deformation in a single plate of the unit cell as shown in Figure 2C, we can intuitively understand the performance using conventional stiffness characterizations from mechanics of materials. The variables that define the dimensions of the unit cell are defined in Figure 2.

If a single thin-walled plate is considered as a beam, the flexural rigidity about $x_{2}$ is far greater than the flexural rigidity about $x_{1}$ if the extruded depth *a* is far greater than the thickness *t*. In addition, if the plate has small thickness relative to its width, it is relatively compliant when a torque is applied about the $x_{3}$ axis. With these beam deformations in mind, we can intuitively see that the unit cell Figure 2B should be able to easily bend about the *x* and *y* axes, but it will resist torsional deformation about *z* with high rigidity (because this would require the individual plates to bend about the $x_{2}$ axis in Figure 2C).

To verify that this intuitive design achieves our purpose, we conducted experiments on 3D printed unit cells and in simulation using the FEA package Nastran In-CAD, a Nastran plug-in for Autodesk Inventor, to determine the mechanical properties of the unit cell. The design dimensions we used in simulation and in physical prototypes are tabulated in Table 1. Six loading cases were conducted to determine the effective flexural rigidities EI, the axial rigidity AE, torsional rigidity GJ and shear rigidities GA of the unit cell as shown in Equation 1.

$$\begin{array}{cc} AE=\frac{F_{z}h}{\delta_{z}}, & GJ=\frac{M_{z}h}{\theta_{z}},\\ {\left(EI\right)}_{x}=\frac{M_{x}h}{\theta_{x}}, & {\left(EI\right)}_{y}=\frac{M_{y}h}{\theta_{y}},\\ {\left(GA\right)}_{x}=\frac{F_{x}h}{\delta_{x}}, & {\left(GA\right)}_{y}=\frac{F_{y}h}{\delta_{y}} \end{array}$$ (1)

**TABLE 1:** Dimensions of unit cell design used in experiments.

Parameter	Value (mm)
Unit cell width, *w*	50
Unit cell height, *h*	12
Extruded depth, *a*	50
Wall thickness, *t*	0.8

**FIGURE 3:**
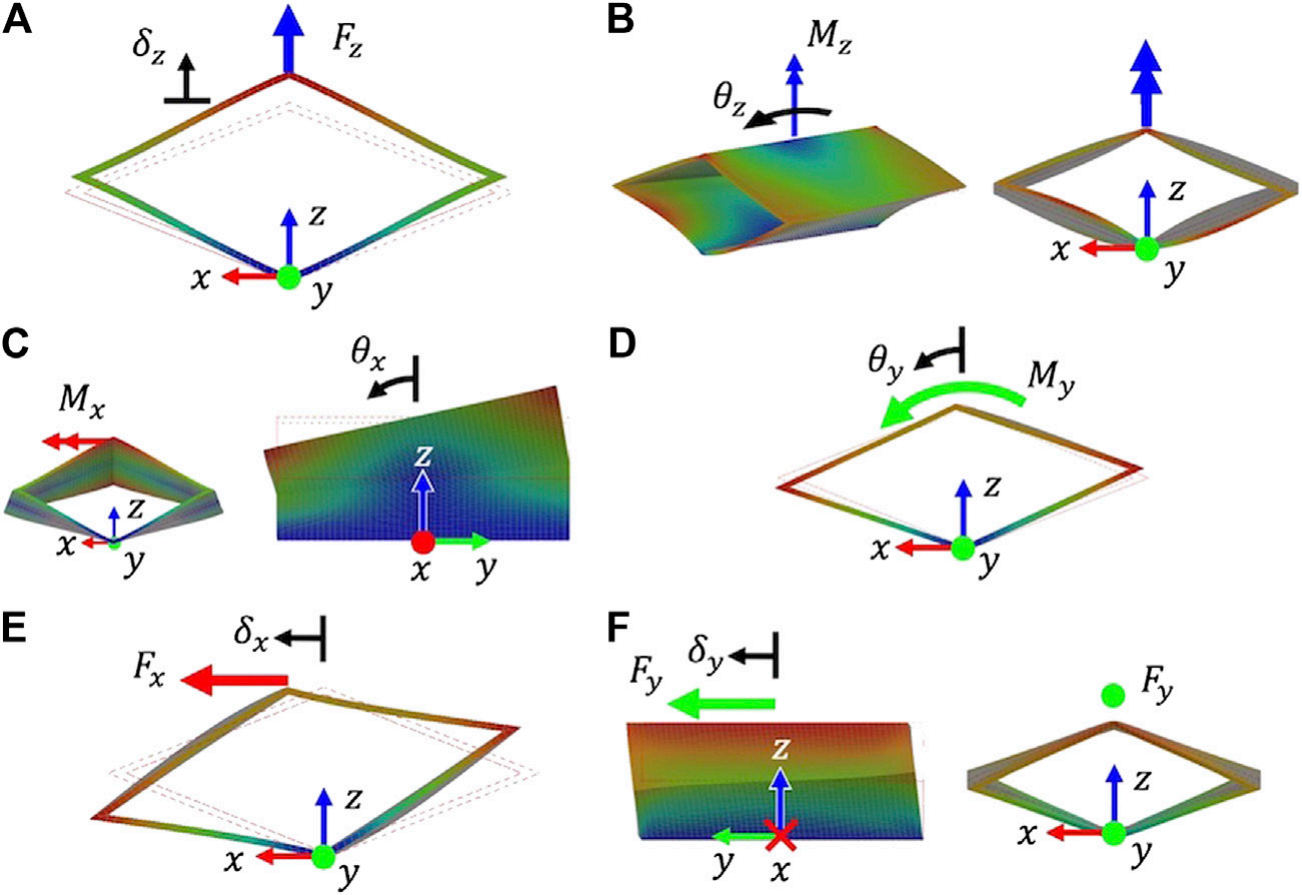
Finite element analysis of a unit cell rhombus with loading cases needed to calculate the axial rigidity **(A)**, torsional rigidity **(B)**, flexural rigidity about *x* **(C)**, flexural rigidity about *y* **(D)**, shear rigidity in *x* **(E)**, and shear rigidity in *y* **(F)**.

The loading conditions and their respective displacements are illustrated in Figure 3. For the axial and flexural tests, the distal vertex of the unit cell is free to rotate and translate. For the torsional test, the distal vertex is constrained from translating in the *z*-direction. For the shear tests, the distal vertex is constrained to only translate in the direction the tip load is applied in; this vertex is also constrained from rotations about the x-,y-, and *z*-axes.

Both FEA and experimental setups assume linear elastic material behavior and small deformation. For a backbone that utilizes multiple unit cells, while the overall structure is capable of large bending angles and translations, the individual unit cells remain in the small deformation regime. For the effective AE, (GA), and ${\left(GA\right)}_{y}$ rigidity values, a linear force $F_{i}$ is applied to the distal end of the unit cell and the resultant displacements $\delta_{i}$ are recorded. For the effective GJ, ${\left(EI\right)}_{x}$, ${\left(EI\right)}_{y}$ rigidity values, a moment $M_{i}$ is applied and the resultant rotations $\theta_{i}$ are recorded. Forces at 0.1 N and moments of 0.005 Nm were applied in the simulation to ensure the small deformation assumption and stay within the linear elastic region. The FEA model in Nastran uses shell elements of parabolic order and the element size was selected to be 1 mm, which utilized 11,022 elements.

Due the anisotropic nature of 3D printing, material properties can vary based on print settings and layer orientation (Dolzyk and Jung, 2019). For our FEA simulations, we calibrate the Young’s modulus *E* of the PETG (MatterHackers PETG 1.75 mm filament) we used to print our unit cells and robot backbone using a three-point flexural test:

$$E=\frac{L^{3}m}{4bd^{3}}$$ (2)

where *L* is the supported length of the beam, *m* is the slope of the load-deflection curve, *b* is the width of the beam, and *d* is the depth of the beam. The PETG sample beam used in the three-point flexural test had a length of 40 mm, a depth/thickness of 0.8 mm and a width of 15 mm. To find the slope of the load-deflection curve, we apply a range of small deflections to the midpoint of the beam using a manual linear rail with an attached digital displacement scale and record the corresponding force readings using a force gauge (Beslands HF-50).

For the experiments with unit cell prototypes, we 3D printed the design specified in Table 1 using MatterHackers PETG on a MakeIt Pro-M 3D printer. The unit cells were fabricated using a nozzle temperature of 245°C and a bed temperature of 60°C. Ultimaker Cura was used to slice the part and allowed us to control the number of wall layers in the part. Because the nozzle diameter of the printer was 0.4 mm, we used two wall layers to achieve our 0.8 mm wall thickness.

**FIGURE 4:**
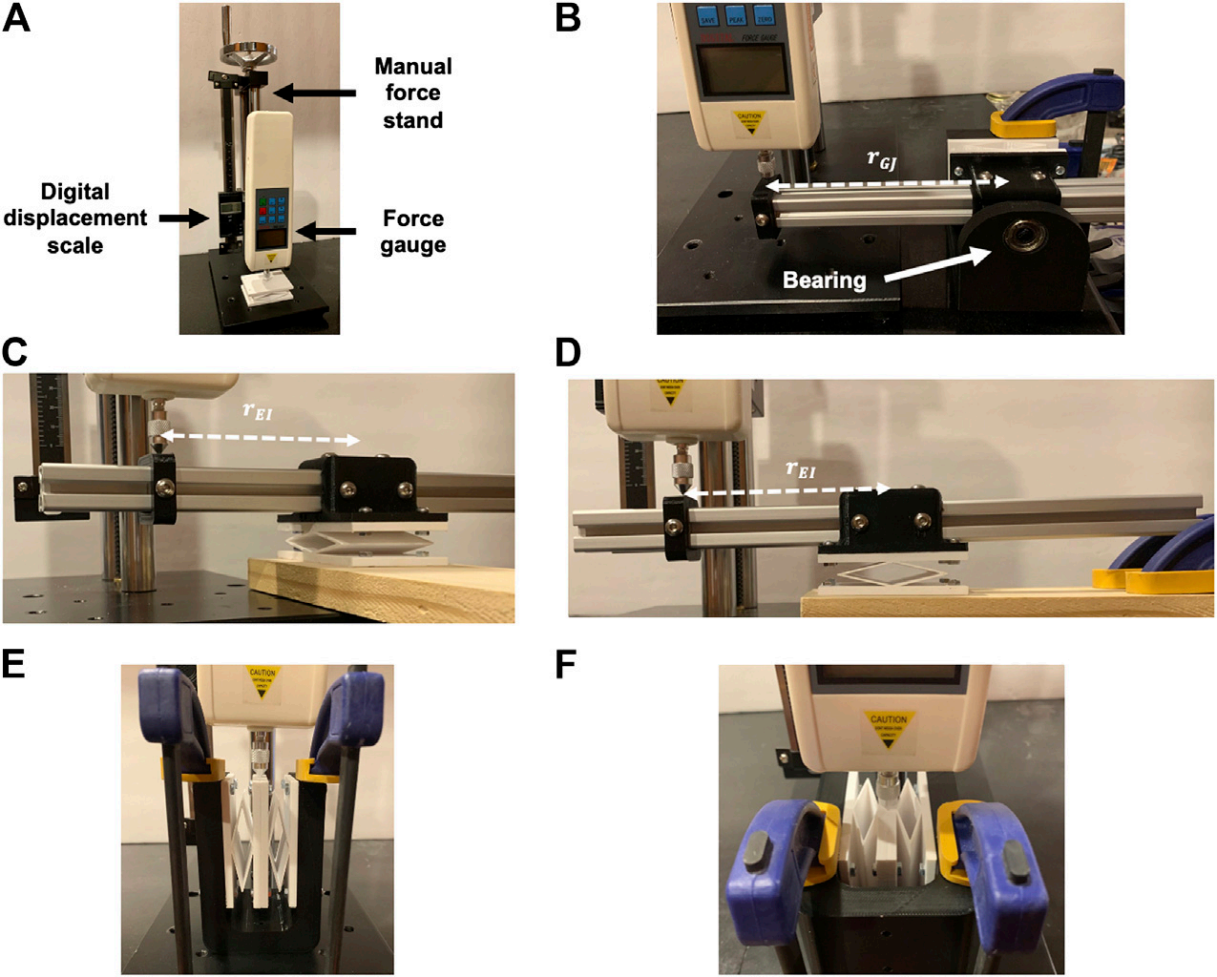
Experimental setups used to determine the effective axial rigidity **(A)**, torsional rigidity **(B)**, flexural rigidity about *x* **(C)**, flexural rigidity about *y* **(D)**, shear rigidity in *x* **(E)**, and shear rigidity in *y* **(F)** of a unit cell design.

The setups used to experimentally determine the mechanical properties of a 3D printed unit cell are shown in Figure 4. A manual force stand, displacement scale and force gauge used in each of the experiments are labeled in experimental setup for determining the effective axial rigidity Figure 4A. This is the same equipment used in the three-point flexural test to calibrate the Young’s modulus of our PETG. For each mechanical property case, the load-displacement relationship is generated by applying a range of deformations to the unit cell and measuring the resultant reaction force using a force gauge (Beslands HF-50). A linear fit is performed for each set of load-displacement data using the method of least squares. The slopes of the linear fit lines correspond to the $F_{i}/\delta_{i}$ and *M*_*i*_/*θ*_*i*_ (where i=x,y,z) in Equation 1. The experimental mechanical properties are then found by multiplying these slopes by the height *h* of the unit cell. To constrain the unit cell to only rotate about its *z*-axis, a bearing mount is attached to the unit cell’s distal plate. For the torsional and flexural rigidity experiments, an 80/20 aluminum extrusion frame is attached to the tip and used as a moment arm for the force gauge. A rectangular cap that is attached to the 80/20 frame is used as a contact point for the force gauge tip and is positioned to set the moment arm distance from the center of the unit cell, which are denoted as $r_{GJ}$ and $r_{EI}$ in Figures 4B–D. The moment arm in the torsional experiment $r_{GJ}$ has a length of 150 mm and the moment arm in both flexural experiments $r_{GJ}$ have a length of 100 mm. The applied moments for the torsional and flexural are computed as $M_{z}=r_{GJ}F_{m}$ and $M_{x}=M_{y}=r_{EI}F_{m}$ where $F_{m}$ is the measured force applied by the force gauge. Using a small angle assumption, these moment arm lengths are also used to calculate the angular deflections $\theta_{i}$ exhibited by the torsional and flexural cases which is simply $\theta_{i}=r_{i}\delta_{m}$ where $\delta_{m}$ is the displacement applied to the 80/20 frame at the moment arm distance location.

For the shear rigidity experiments, two unit cells are attached together at their end plates. The base plate of the two unit cells are fixed within in a U-shaped jig as shown in Figures 4E,F. Applying a force at the midpoint between the attached two unit cells plates enforces translation in the direction of force without additional rotation or translation deflections. It should be noted that because two unit cells are connected in parallel for the shearing experiments, the resulting load-deflection data would correspond to the shear rigidity of two unit cells. The effective shear rigidity values ${\left(GA\right)}_{x}$ and ${\left(GA\right)}_{y}$ for a single unit cell are simply half the shear rigidity values of the two combined unit cells.

For the backbone prototype, we used 13 repeated unit cells of equal dimension as shown in Figure 2A, resulting in a backbone bounded by a 50 × 50 × 165 mm rectangular prism. The repeated unit cell dimensions are tabulated in Table 1. Using an Ohaus CS 5000 weight scale, the mass of the backbone was measured to be 73 g (without actuation rods inserted). Based on the fatigue analysis of 3D printed PETG, our backbone should be able to withstand at least 7,500 compression cycles before failure or fracture, a sufficient fatigue life for conducting our experiments (Dolzyk and Jung, 2019).

### 2.2 Actuation Design for Tip Load Resistance

**FIGURE 5:**
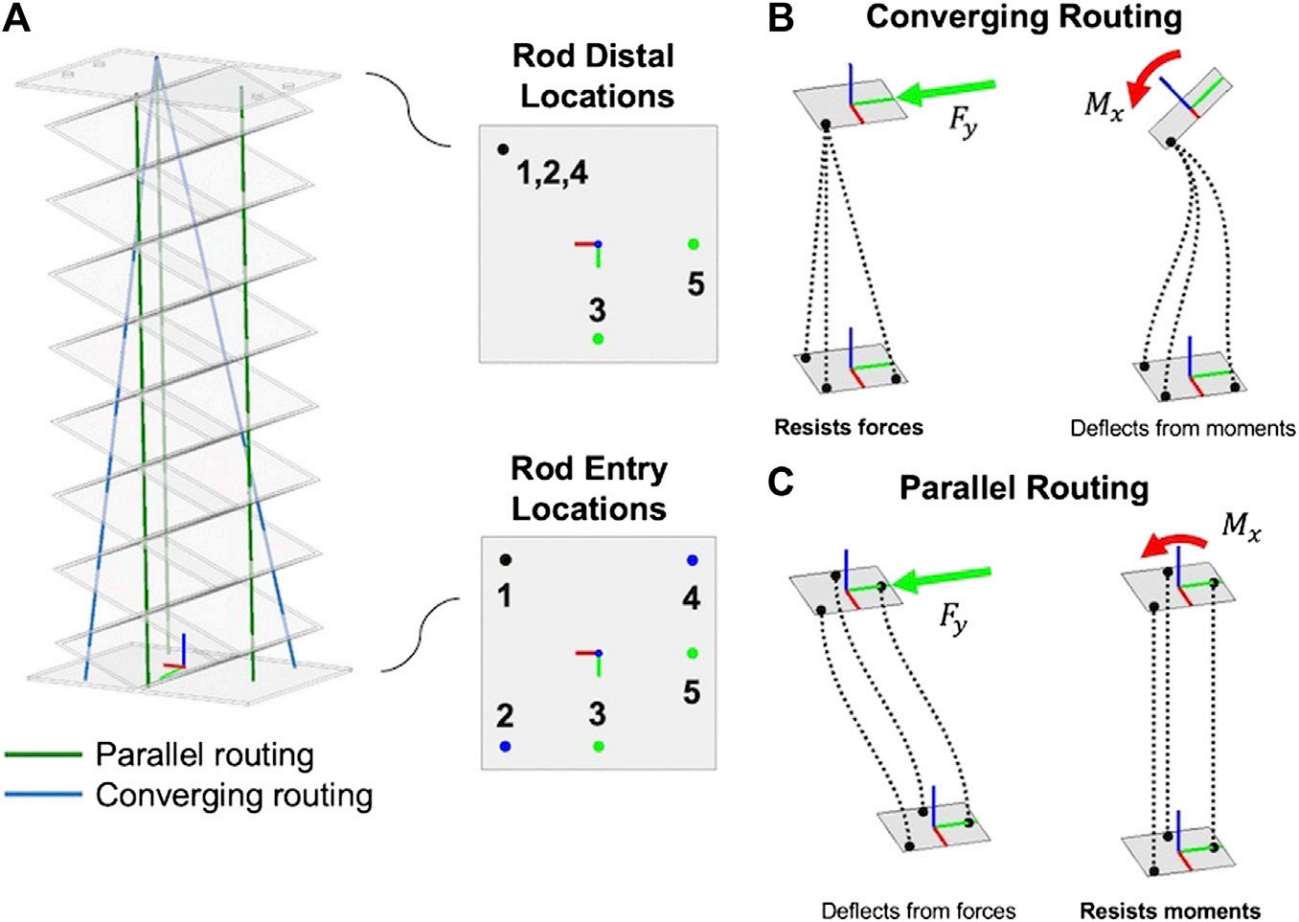
Prototype robot actuation pathway **(A)**, which utilizes **(B)** converging actuation rod routing, which enables strength against tip forces end-effector and **(C)** traditional parallel actuation rod routing that enables strength against tip moments.

While having a backbone with high torsional rigidity can greatly improve its payload capacity, the design of the actuator routing paths can also be used to increase output stiffness. For our robotic prototype, we used a combination of parallel and convergent actuator routing as shown in Figure 5A. Rods 2, and 4 use converging routing and rods 3 and 5 use parallel routing; rod 1 belongs to both converging and parallel routing sets. Using both types of routing modifies 1) the stiffness of the robot and 2) the end effector degrees of freedom.

As shown in Figure 5C, continuum robots with parallel actuator routing exhibit an s-shaped deformation mode when a transverse tip force is applied to the end-effector. However, the parallel actuator routing resists moment loads with high stiffness. Conversely, as shown in Figure 5B robots with converging routing paths exhibit deformation when tip moments are applied, but resist direct tip force loading with high stiffness because the non-parallel actuation forces (tension or compression) are capable of producing a resultant in the opposite direction of the tip load. Thus, using both converging and parallel actuation routing simultaneously provides high stiffness with respect to both forces and moments at the tip.

**FIGURE 6:**
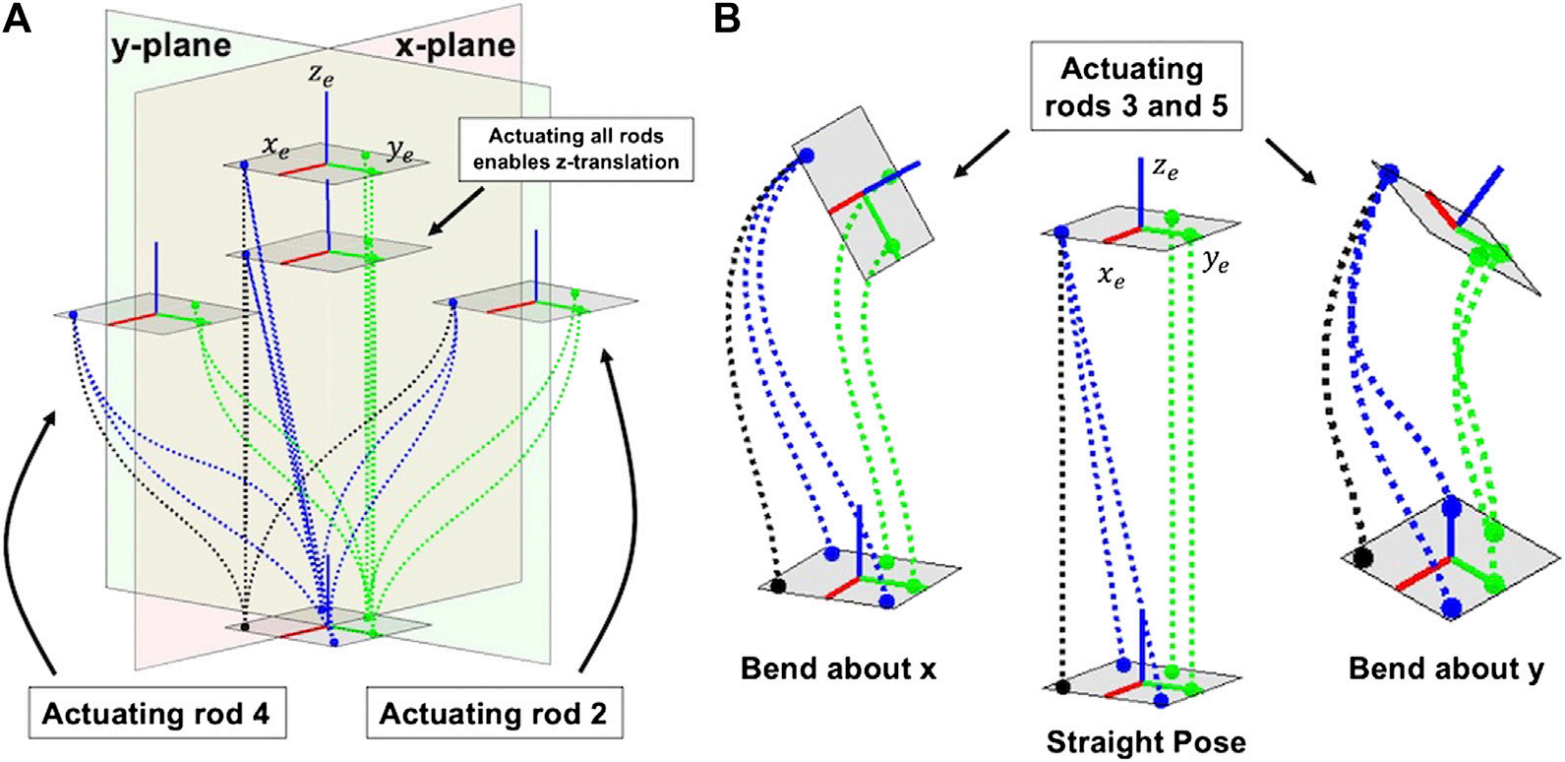
Converging actuation rods enable translation of the tip **(A)** and parallel actuation routing enable bending at the tip **(B)**. The dashed lines represent the actuation rods, with the green rods representing the parallel routing rods (3 and 5), the blue representing the converging routing rods (2 and 4) and the black dashed line is rod 1, which acts in both parallel and converging routing.

If the backbone has a high torsional rigidity that is much greater than its flexural rigidities, this also means that the angular twist degree of freedom about the robot’s $z_{e}$ axis will be constrained. Therefore, only five actuation rods are necessary to control the remaining five degrees of freedom - translations in *x*,*y*, and *z* and angles about the *x*- and *y*-axes. The converging actuation rods enable three translation motions of the tip while the parallel actuation rods enable two bending motions at the tip as shown in Figure 6. We should note that this is a similar approach to a freedom and constraint analysis (Hopkins and Culpepper, 2011).

If the actuation rods are able to withstand both compression and tension, then the robot can achieve symmetric motions within its workspace. Furthermore, our actuation routing is designed such that none of the actuation rods pass through the centerline of the backbone. This allows inner channels to be created within the backbone which would allow devices at the end-effector such as grippers, sensors or cameras to have their cables pass through the backbone.

### 2.3 Robot Embodiment

**FIGURE 7:**
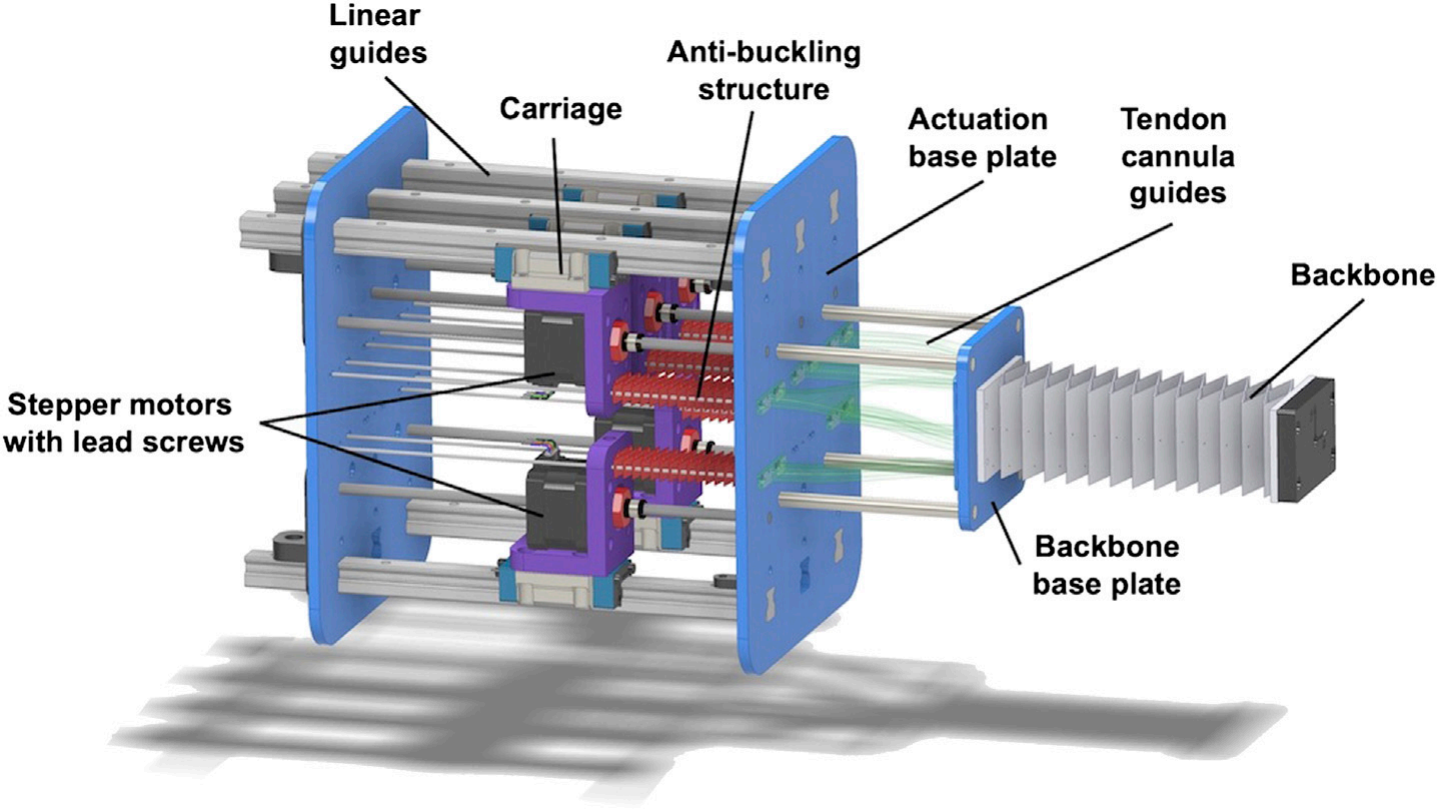
Actuation platform used for experiments with each component labeled.

We designed and fabricated an actuation setup to experimentally test our design paradigm for leveraging backbone and actuation routing geometry. There are four sections of the robot: 1) the rhombus backbone, 2) the cannula guides, 3) the anti-buckling structures, and 4) the actuation pack assembly. The complete actuation setup and details of its sections are shown in Figure 7. The rhombus backbone is fabricated using PETG and consists of 13 repeated unit cells with the same cell dimensions as tabulated in Table 1. We use NEMA 17 non-captive linear stepper actuators (PBC Linear) to translate the actuation rods, which are 0.024” (0.61 mm) diameter spring-steel rods (ASTM A228). Relative to the mechanical properties of our backbone, the actuation rods contribute an insignificant amount of bending stiffness to the structure.

#### 2.3.1 Cannula Guides

**FIGURE 8:**
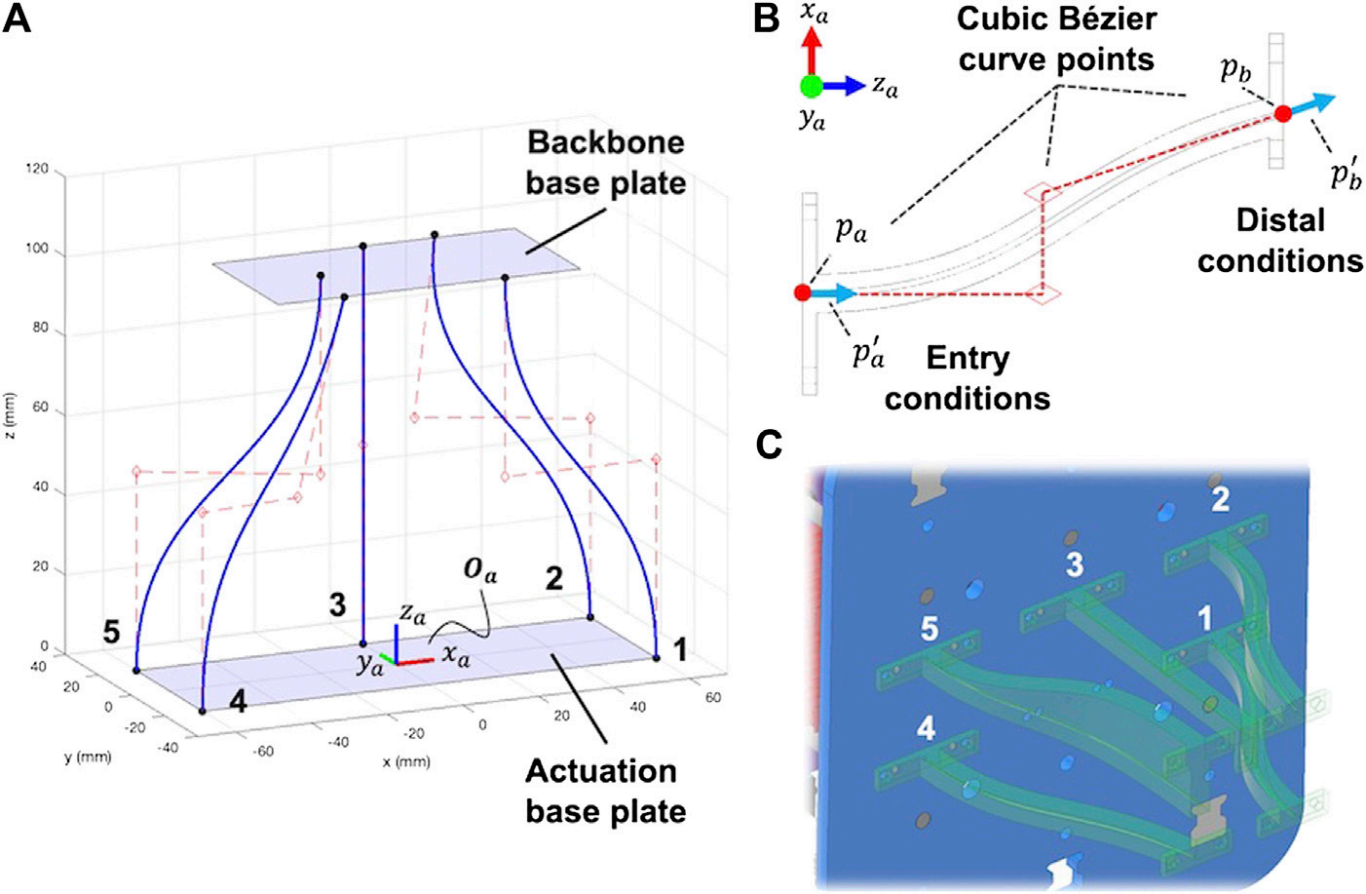
Cannula guides designed using cubic Bézier curves **(A)** that are generated from specifying entry and distal conditions **(B)**. Cannula guides are printed individually and attached to the actuation and backbone base plates **(C)**.

To achieve the parallel and convergent actuation routing paths that run in straight lines from the base of the backbone to the tip, we designed a set of curved rigid cannula guides that the actuation rods pass through on their way from the pack of linear actuators to the backbone. These rigid cannula guides solve three challenges: 1) they allow converging actuation rods 2 and 4 to enter into the backbone at their prescribed angles, 2) they prevent rod buckling between the actuation base plate and the backbone base plate, and 3) they allow the linear actuators to be spaced without interference. We used cubic Bézier curves to design the guides to satisfy the point-slope entry and distal conditions. Figure 8A shows the Bézier curves that route each actuation rod through the actuation base plate and into the backbone base plate where the tendons are then inserted through the backbone. We designed each curve by specifying the entry and distal point and slope conditions for each rod as shown in Figure 8B. The entry conditions for a single actuation rod consist of the entry location on the actuation base plate $p_{a}$ in the $O_{a}$ reference frame and the entry slope $p_{a}^{\prime }$; for each rod, the entry slope condition is always straight i.e. $p_{a}^{\prime }=\left[0,0,0\right]$. The distal condition $p_{b}$ specifies the rod entry locations into the backbone base plate (in the $O_{a}$ frame) and $p_{b}^{\prime }$ specifies the slope of the rod as it enters the backbone base plate.

Each cannula guide was printed using Hatchbox PLA filament. Polytetrafluoroethylene (PTFE) tubing (18 gauge) was inserted within each of the cannulas to reduce the friction between the steel rods and the PLA walls. As shown in Figure 8C, the cannula guides for rods 2 and 5 were created with additional material sections to accommodate 3D printing without supports.

#### 2.3.2 Anti-Buckling Structure

2.3.2

**FIGURE 9:**
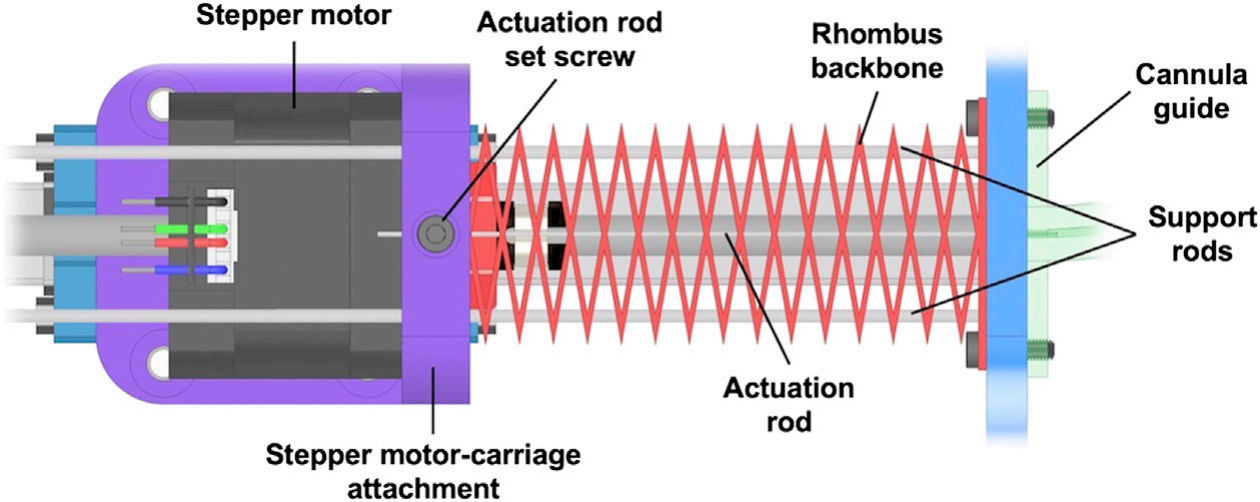
Detailed design of the anti-buckling structure used to enable compressive actuation rod loads.

To allow for push-pull actuation, there is a free length of the actuation rods between the stepper motor carriage and the actuation base plate. To prevent the rods from buckling in this region before entering the cannula guides, we utilized a smaller version of our rhombic backbone structure, 3D printed with Hatchbox PLA filament, to create an anti-buckling mechanism between the actuators and the robot backbone. The schematic of this diagram is found in Figure 9. The mechanism consists of a small-scale version of the rhombus backbone that allows the actuating rod to pass through the center of the structure. Two 2 mm diameter steel rods are routed through the miniature backbone structure to increase its resistance to buckling when in compression. These support rods are fixed between the plates attached to the backbone structure and the actuator base plate.

### 2.4 Experimental Compliance Analysis

To assess the strength performance of our robotic prototype, we experimentally determined the end-effector compliance matrix in (1) straight, 2) translation in x, 3) translation in y, 4) bending about x and 5) bending about y. The compliance matrix relates the applied force at the end-effector **F** and its resultant displacement Δ **p** in the global reference as follows:

$$\begin{array}{l} \Delta p=CF\\ \left[\begin{array}{c} \text{Δ}p_{x}\\ \text{Δ}p_{y}\\ \text{Δ}p_{z} \end{array}\right]=\left[\begin{array}{ccc} C_{11} & C_{12} & C_{13}\\ C_{21} & C_{22} & C_{23}\\ C_{31} & C_{32} & C_{33} \end{array}\right]\left[\begin{array}{c} F_{x}\\ F_{y}\\ F_{z} \end{array}\right] \end{array}$$ (3)

**FIGURE 10:**
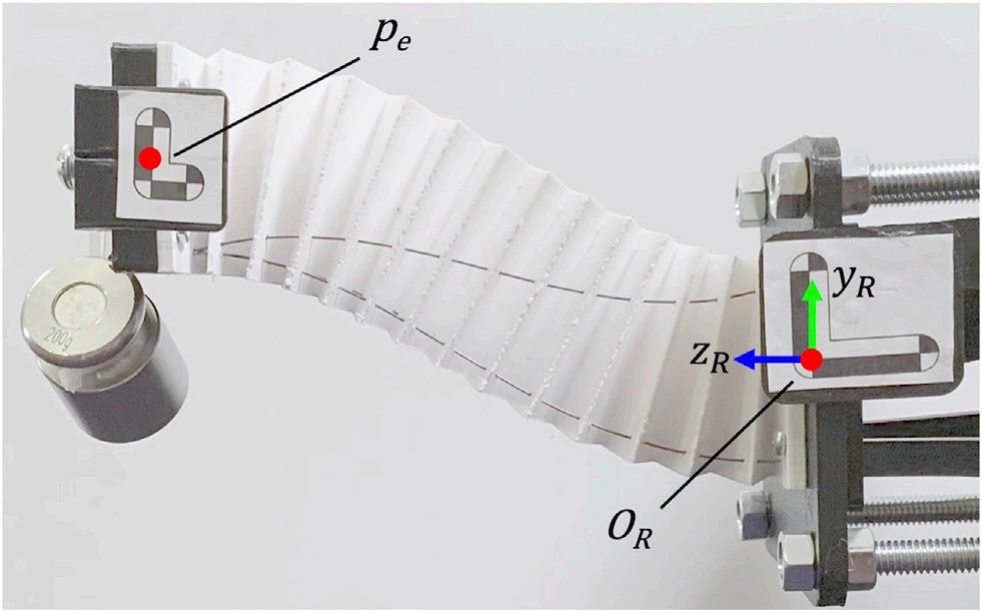
Experimental setup for calculating the compliance matrix for each pose. The ‘Translation in Y’ pose is shown with a 200 g pre-load attached to the end-effector that is applied in the negative *y* direction in $O_{R}$.

**Figure 11:**
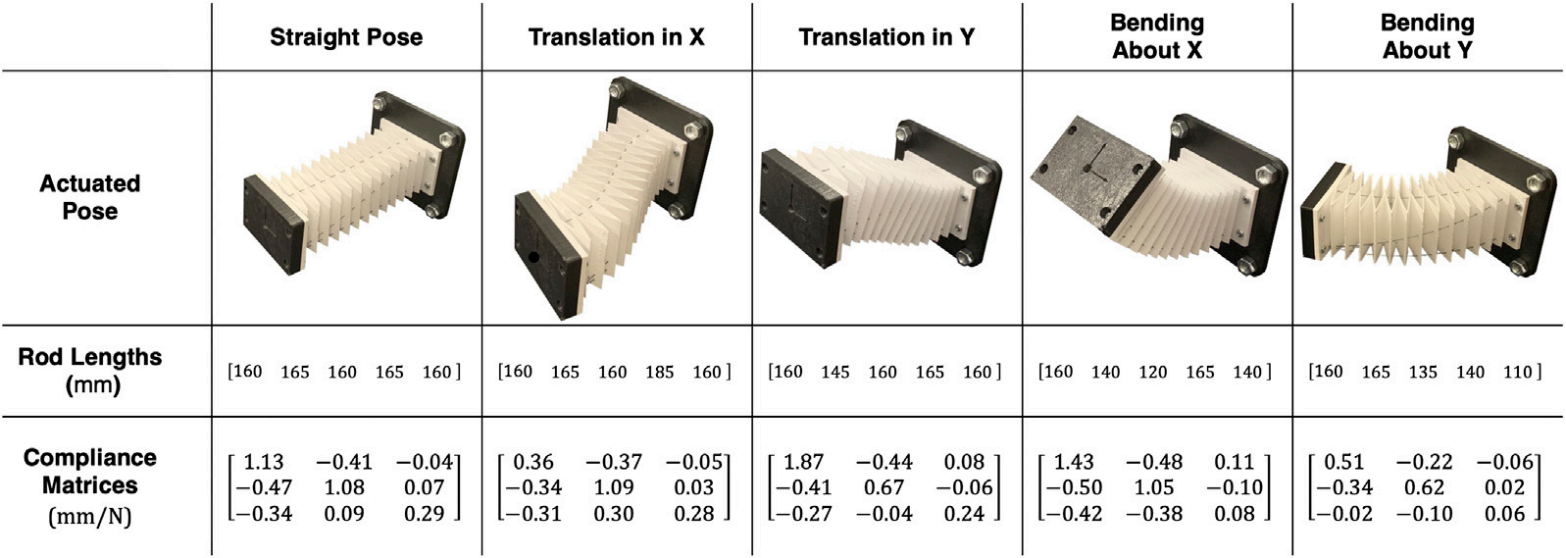
Candidate robot poses used in compliance analysis. The actuation rod length configurations necessary to achieve the pose and resultant compliance matrix for that pose are shown below.

To calculate the compliance matrix for each pose, we apply end-effector tip loads in the *x*, *y*, and *z* directions relative to the base frame of the robot $O_{R}$ as shown in Figure 10 and measure the change in tip position using a stereoscopic camera (MicronTracker H3-60). Markers are attached to the distal end and base of the robot to measure the deflections at the end-effector (in the base frame of the robot $O_{R}$). For each loading case, we apply a pre-load of 200 g to mitigate the backlash that can occur in the actuation setup and record the marker positions and orientations after loading. We then apply an additional 200 g at the tip (total of 400 g tip load) and record the resultant marker positions after this loading. The displacements from when the robot is loaded with 200 g to when it’s loaded with 400 g are used for $\text{Δ}p_{x}$, $\text{Δ}p_{y}$ and $\text{Δ}p_{z}$. This process is performed for the five pose cases as shown in Figure 11.

## 3 Results and Discussion

### 3.1 Backbone Mechanical Properties

**Figure 12:**
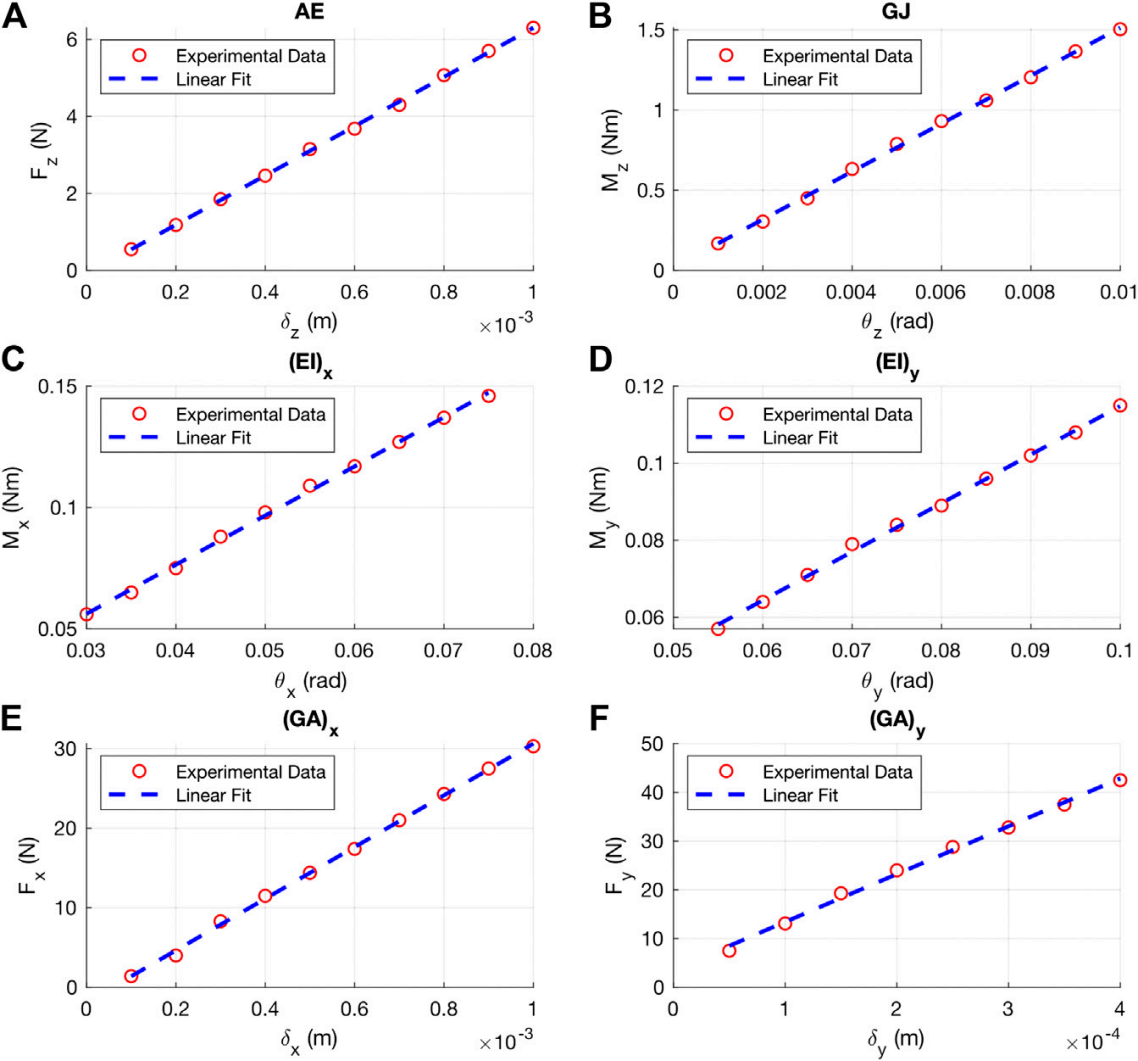
Experimental load-deflection lines used to calculate the axial rigidity **(A)**, torsional rigidity **(B)**, flexural rigidity about x **(C)**, flexural rigidity about y **(D)**, shear rigidity in x **(E)** and shear rigidity in y **(F)**. of a 3D printed PETG unit cell.

For the flexural test used to determine the Young’s modulus of the PETG we used in experimental properties, a linear fit of the load-deflection resulted in a slope of 585 N/m. Using Equation 2, the Young’s modulus of our PETG is calculated as 1.22 GPa, similar to other reported 3D printed PETG (Dolzyk and Jung, 2019). The load-deflection lines for each mechanical property case are show in Figure 12.

The experimental load-displacement lines for each mechanical property is shown in Figure 12. The $R^{2}$ of the linear fit for each case is 0.99, confirming that our applied deformations were within the linear elastic material range. Using the slopes of these linear fits, the mechanical properties calculated using Equation 1 and the corresponding FEA and experimental results are tabulated in Table 2. Note that the load-deflection data in Figures 12E,F is for two unit cells connected; the slope of this fit is reduced by half to determine ${\left(GA\right)}_{x}$ and ${\left(GA\right)}_{y}$ for a single unit cell. The experimental torsional rigidity is greater than the flexural rigidities ${\left(EI\right)}_{x}$ and ${\left(EI\right)}_{y}$ by factors of approximately 75 and 119 respectively. Additionally, we can see that the experimental shear rigidities ${\left(GA\right)}_{x}$ and ${\left(GA\right)}_{y}$ are approximately 2.53 and 7.65 times greater than the axial rigidity AE. For comparison, a rod with a simple circular cross section has torsional rigidity of just 1/(1+ν) times its flexural rigidity, and a shear rigidity of 1/[2(1+ν)] times its axial rigidity, where *ν* is Poisson’s ratio of the example backbone. If this rod was made of steel (ν=0.3), the torsional rigidity would only be 0.77 times its flexural rigidity and the shear rigidity would only be 0.38 times its axial rigidity.

**TABLE 2:** Mechanical properties of unit cell from FEA and experimental.

Unit cell mechanical property	FEA value	Experimental value
Axial rigidity, AE	77 N	77 N
Torsional rigidity, GJ	4.68 Nm^2^	1.79 Nm^2^
Flexural rigidity about x, ${\left(EI\right)}_{x}$	0.029 Nm^2^	0.024 Nm^2^
Flexural rigidity about y, ${\left(EI\right)}_{y}$	0.016 Nm^2^	0.015 Nm^2^
Shear rigidity in x, ${\left(GA\right)}_{x}$	178 N	195 N
Shear rigidity in y, ${\left(GA\right)}_{y}$	7,600 N	589 N

FEA, finite element analysis.

For AE, ${\left(EI\right)}_{x}$, ${\left(EI\right)}_{y}$ and ${\left(GA\right)}_{x}$, the FEA simulation provided values that are in close agreement with experiment. While the FEA simulation predicted that the torsional rigidity GJ and shear rigidity ${\left(GA\right)}_{y}$ to be much greater than what was experimentally determined, the discrepancy is likely due to small deflections in the support fixtures for these experiments. Furthermore, both FEA and experiments confirm that the stiffness of these modes is very high, so that continuum robot deformations will be primarily caused from bending and axial deformation, which was our design goal. These experiments confirm our assumptions that the rhombus unit cell design can enable a backbone design that has high torsional rigidity relative to its flexural rigidity. In addition, when using calibrated material properties, FEA can be used as a design tool to achieve a rhombus backbone design with desired mechanical properties.

### 3.2 Experimental Compliance Results

Figure 11 contains the results of the experimentally determined compliance matrices for each of the five poses. We can see that the robot exhibits low compliance at the tip for all configurations. From both inspection of the robot loading scenario and the compliance matrix results, we do not see the traditional s-shape mode of deformation found in near-straight poses. We also do not see significant torsional deformation in the bending cases with out-of-plane loads.

### 3.3 Payload Task Demonstration

**FIGURE 13:**
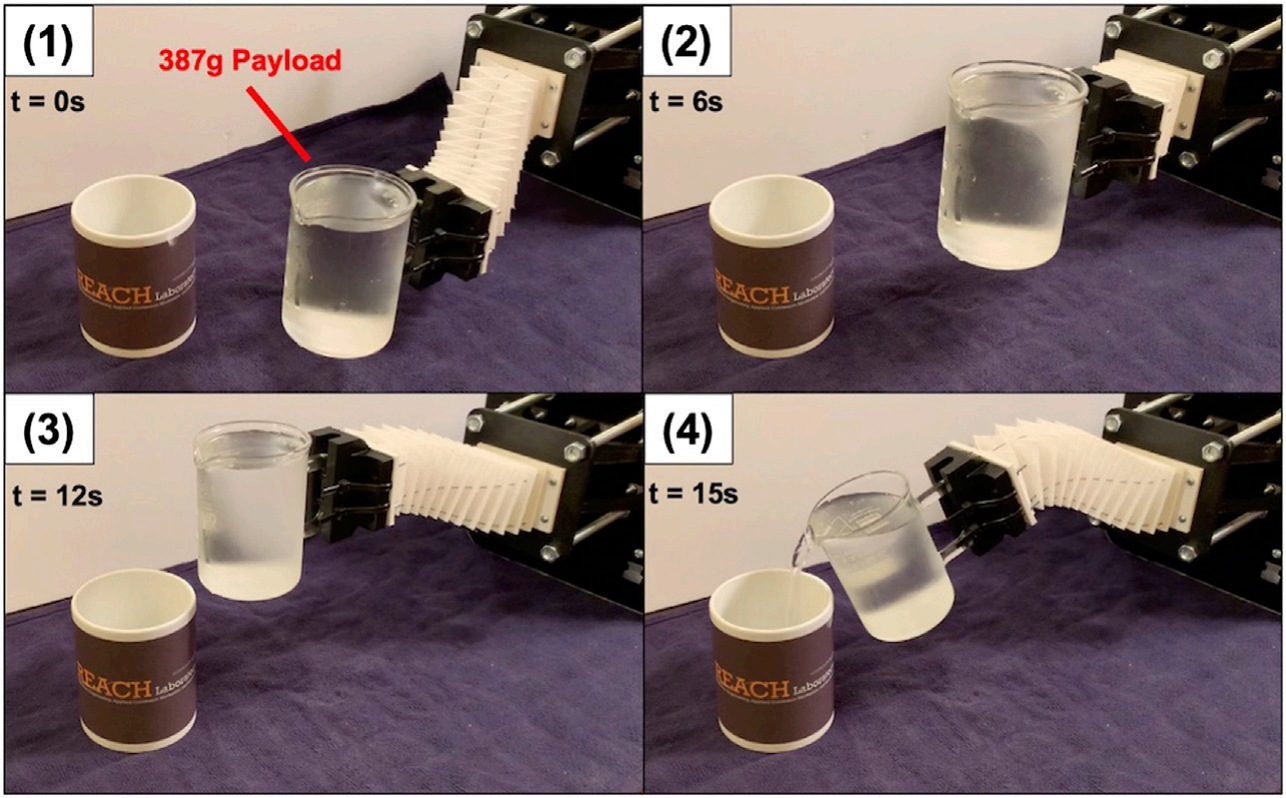
Payload carrying demonstration with time stamps.

Inspired by the results of our compliance experiments, we demonstrated the robot’s load capacity by carrying a 400 g to a desired location. Figure 13 shows the timestamps of the carrying task. In this demonstration, the robot is loaded at its end-effector with the payload. The cup assembly without any water added is 160 g. 8oz of water is added (227 g) to the cup assembly for a combined 387 g tip load (stage 1). Then, the robot lifts the payload in the positive *y*-direction (stage 2) and then translates in the negative *x*-direction (stage 3). Finally, the end-effector smoothly rotates to pour the water into the coffee mug on the table (stage 4). This task was accomplished by actuating a single motor at a time within 15 s. No oscillations were observed, and none of the water spilled from the cup assembly while in transit.

### 3.4 Discussion and Future Work

The results of the compliance characterization and carrying demonstrations show how a strong continuum robot can be developed out of common materials by leveraging the geometric design improvements for the backbone and actuation rod routing without introducing additional stiffening components or weight into the robot design. In our future work, we will investigate fabrication techniques that will enable us to manufacture these robot designs at minimally-invasive surgical scales as well as at large scales for human-robot collaboration tasks. In our FEA model and 3D printed unit cells, we did not use design features such as fillets at the vertices. However, using fillets in future designs could greatly reduce stress concentrations. Developing a mechanics-based model that relates the geometry of the unit cell to the effective resultant rigidities of the backbone would be useful for design and optimization of the backbone structure via adaptation of general Cosserat rod kinematic models (Rucker and Webster III, 2011). We anticipate that the kinematics of this robot could be modeled as a parallel continuum robot in which the backbone and tendons could be modeled as Cosserat rods (Black et al., 2018; Orekhov et al., 2017).

## Data Availability

The raw data supporting the conclusions of this article will be made available by the authors, without undue reservation.
